# Prognostic value of the chest X-ray in patients hospitalised for heart failure

**DOI:** 10.1007/s00392-021-01836-9

**Published:** 2021-03-22

**Authors:** Daniel Pan, Pierpaolo Pellicori, Karen Dobbs, Jeanne Bulemfu, Ioanna Sokoreli, Alessia Urbinati, Oliver Brown, Shirley Sze, Alan S. Rigby, Syed Kazmi, Jarno M. Riistama, John G. F. Cleland, Andrew L. Clark

**Affiliations:** 1 Department of Respiratory Sciences, University of Leicester, University Road, Leicester, LE1 7RH UK; 2 Castle Hill Hospital, Castle Road, Cottingham, HU16 5JQ UK; 3 Robertson Centre for Biostatistics, Boyd Orr Building, University of Glasgow, Glasgow, G12 8QQ UK; 4 Philips Research, High Tech, Campus 34, 5656 AE Eindhoven, The Netherlands; 5 Department of Cardiovascular Sciences, University of Leicester, University Road, Leicester, LE1 7RH UK; 6 Hull York Medical School, University of York, John Hughlings Jackson Building, University Road, Heslington, YO10 5DD York UK; 7 University of Hull, Cottingham Road, Hull, HU6 7RX UK; 8 Philips Image Guided Therapy, Veenpluis 4-6, 5684 PC Best, The Netherlands

**Keywords:** Chest radiograph, Acute heart failure, Mortality, Congestion

## Abstract

**Background:**

Patients admitted to hospital with heart failure will have had a chest X-ray (CXR), but little is known about their prognostic significance. We aimed to report the prevalence and prognostic value of the initial chest radiograph findings in patients admitted to hospital with heart failure (acute heart failure, AHF).

**Methods:**

The erect CXRs of all patients admitted with AHF between October 2012 and November 2016 were reviewed for pulmonary venous congestion, Kerley B lines, pleural effusions and alveolar oedema. Film projection (whether anterior–posterior [AP] or posterior–anterior [PA]) and cardiothoracic ratio (CTR) were also recorded. Trial registration: ISRCTN96643197

**Results:**

Of 1145 patients enrolled, 975 [median (interquartile range) age 77 (68–83) years, 61% with moderate, or worse, left ventricular systolic dysfunction, and median NT-proBNP 5047 (2337–10,945) ng/l] had an adequate initial radiograph, of which 691 (71%) were AP. The median CTR was 0.57 (IQR 0.53–0.61) in PA films and 0.60 (0.55–0.64) in AP films. Pulmonary venous congestion was present in 756 (78%) of films, Kerley B lines in 688 (71%), pleural effusions in 649 (67%) and alveolar oedema in 622 (64%).

A CXR score was constructed using the above features. Increasing score was associated with increasing age, urea, NT-proBNP, and decreasing systolic blood pressure, haemoglobin and albumin; and with all-cause mortality on multivariable analysis (hazard ratio 1.10, 95% confidence intervals 1.07–1.13, *p* < 0.001).

**Conclusions:**

Radiographic evidence of congestion on a CXR is very common in patients with AHF and is associated with other clinical measures of worse prognosis.

**Graphic abstract:**

**Figure:**
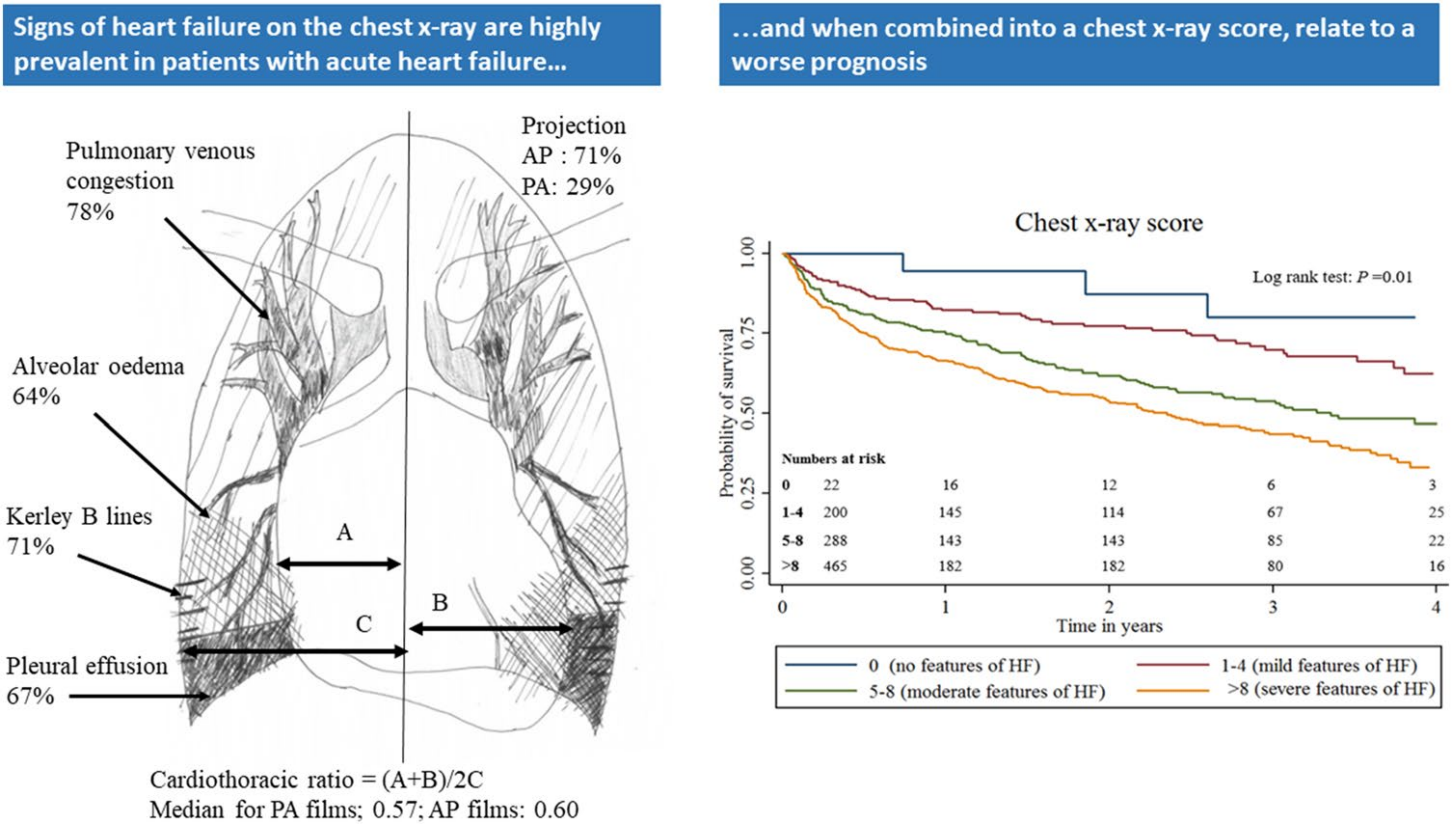
Signs of heart failure are highly prevalent in patients presenting to hospital with acute heart failure and when combined into a chest x-ray score, relate to a worse long term risk of death

## Introduction

Heart failure (HF) is the leading cause of hospital admission in people aged 65 years or older in developed countries [1]. The chest X-ray (CXR) is one of the core investigations of breathless patients [2, 3]. In 1917, Danzer first identified cardiomegaly as a possible indicator of left ventricular dilation [4]. As hydrostatic pressure increases in the lungs, signs of pulmonary congestion start to appear—pulmonary venous congestion, interstitial oedema, alveolar oedema and pleural effusions—that are often used as entry criteria in trials of therapies for patients presenting with acute HF [5, 6].

The CXR is not a diagnostic test for HF. There is little relation between cardiothoracic ratio (CTR) and left ventricular systolic function [7]. Not all patients with acute HF have pulmonary congestion on their CXR [8]. However, amongst patients presenting to hospital with acute myocardial infarction, worsening features of HF on the CXR are related to increasing risk of death [9–13].

Fifty years ago, when rheumatic heart disease was common, acute pulmonary oedema was dramatic and life threatening [14]. Today, most patients admitted to hospital with HF no longer have new acute pulmonary oedema but instead present with decompensation of chronic underlying ventricular dysfunction, as a consequence of gradual but progressive increases in cardiac filling pressures [15, 16]. We, therefore, described the appearance of the initial CXR in a modern cohort of patients hospitalised for HF and assessed the relation between CXR appearance and outcome. We constructed a CXR scoring system and assessed the relation between the score and outcome in addition to clinical variables.

## Methods

### Study setting

The observational study to predict readmission for heart failure patients (OPERA-HF) is a prospective observational study which enrolled patients hospitalised for HF in the Hull University Hospitals NHS Trust, UK. Patients had to fulfil the following criteria to be included: age > 18 years; usual residence in the region served by the Hull University Hospitals NHS Trust; hospitalisation with HF; treatment with loop diuretics; and at least one of the following; left ventricular ejection fraction ≤ 40%; left atrial dimension ≥ 4.0 cm [17], or N-terminal pro-B-type natriuretic peptide (NT-proBNP) > 400 pg/mL (if in sinus rhythm) or > 1200 pg/mL (if in atrial fibrillation—AF) [18]. Patients who were unable to understand and comply with the protocol or unable or unwilling to give informed consent were not included in the study.

### Chest radiography

**Fig. 1:**
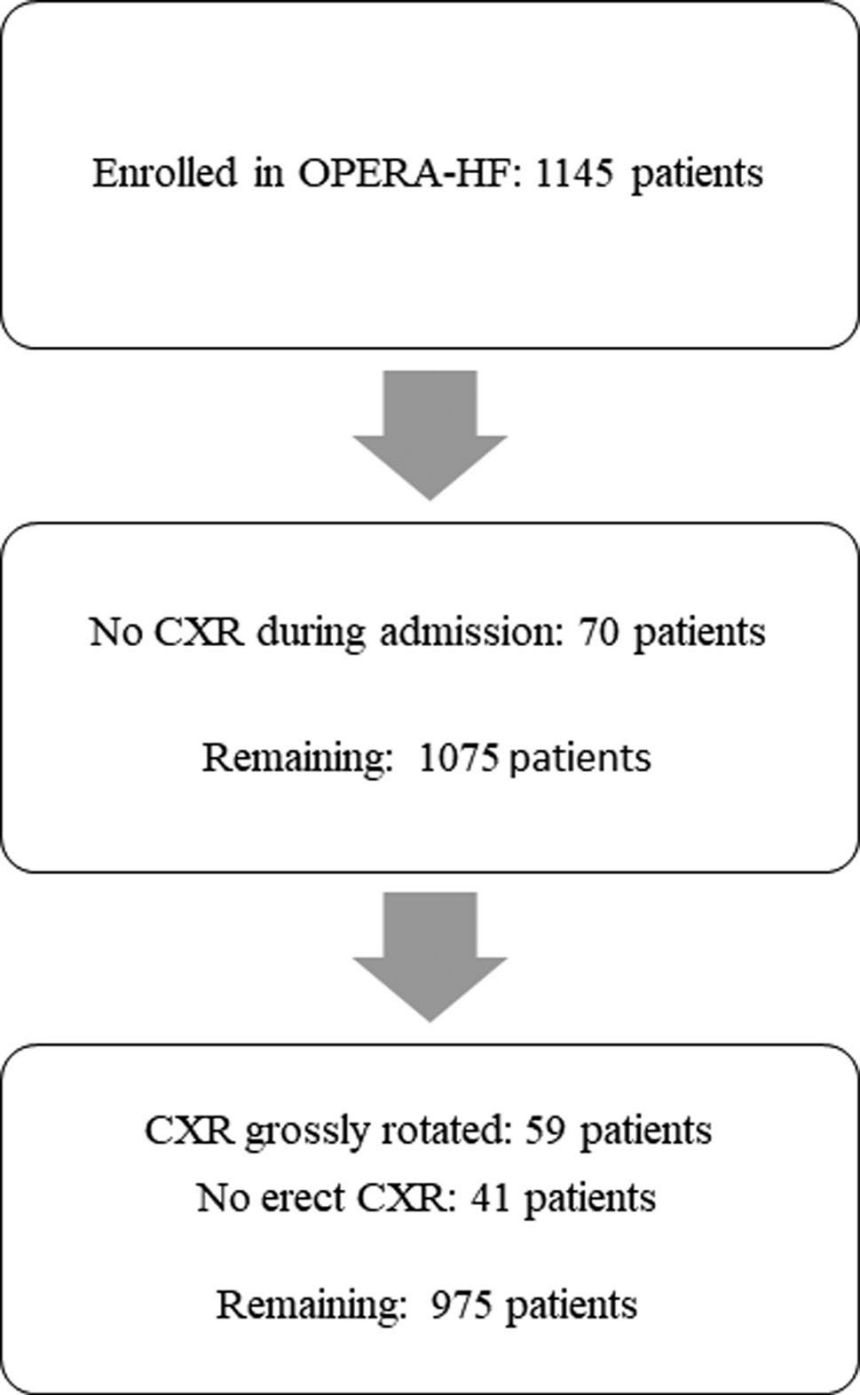
Flowchart illustrating the flow of patients through the study. *CXR* chest X-ray, *CT* computed tomography

The flow of patients through the study is shown in Fig. 1. The position (supine or erect) and projection of each film is written physically on the X-ray. In the present analysis, we included all patients who had an erect chest radiograph. The patient’s first radiograph on admission was reviewed. We excluded patients who had had only a supine film (as fluid in the lungs and pleura might have been distributed differently) or in whom the film was too rotated for interpretation. One investigator (Daniel Pan, DP) retrospectively reviewed all CXRs, grading the presence and severity of potential features of HF, blind to all other clinical data.

**Fig. 2:**
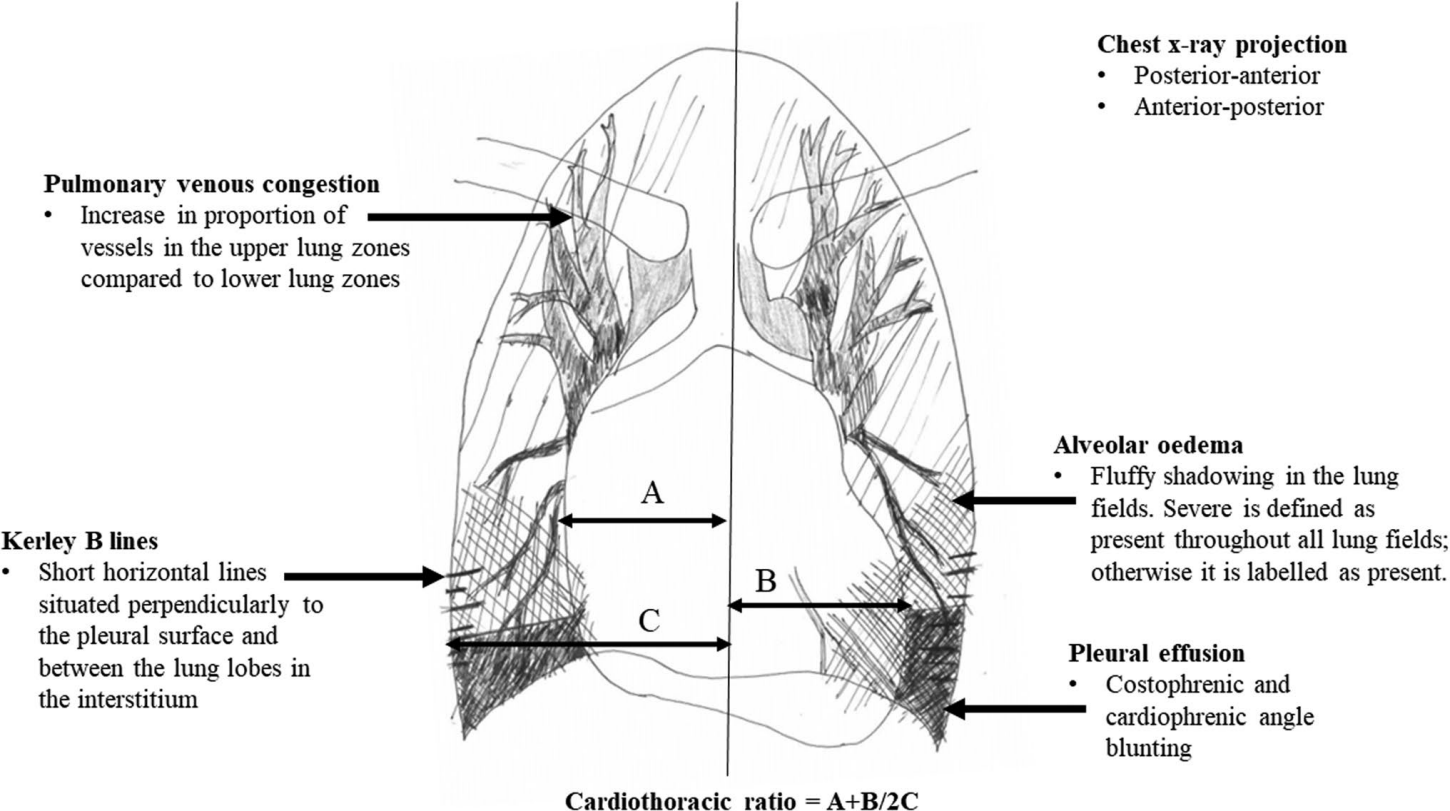
Illustration demonstrating how chest X-ray features of heart failure was identified

The approach to grading the CXR is illustrated in Fig. 2. The following were recorded for each patient:

- The film projection—whether posterior–anterior (PA) or anterior–posterior (AP);
- Alveolar oedema—graded as absent, present (oedema covering any of the lungs, but not all zones in both lungs) or severe (oedema covering all zones in both lungs).
- Kerley B lines—graded as the presence or absence of prominent interstitial fissure lines in and between the lung lobes.
- Pleural effusions—graded as present or not present. Presence is when there are both costophrenic and cardiophrenic blunting of one lung field.
- Pulmonary venous congestion—graded as present or absent. Presence is when there is an increase in the proportion of vessels in the upper lung zones compared to the lower lung zones.
- Heart size—measured as CTR.

The CTR was calculated using the original method described by Danzer [4]. A single line is drawn dividing the cardiac silhouette vertically through the midline. Measurement ‘A’ is widest point from the right heart border to the line; measurement ‘B’ is the widest point from the left heart border to the line. Measurement ‘C’ is the widest measurement from the midline to the inner borders of the ribs. Thus, the CTR is defined as (A + B)/2C. An unmeasurable CTR was recorded where the cardiac borders were too obscure for accurate measurement. Although modern dictum suggests that accurate CTR interpretation is not possible in AP films, we considered a CTR of > 0.55 to be cardiomegaly regardless of projection, in keeping with the previous literature [19].

Signs related to other pathology as routinely reported by the radiologist were also recorded. To assess repeatability of findings, 25 CXRs were randomly selected and scored by a second investigator (Shirley Sze, SS).

Patients were followed until 1st of May 2017; the main outcome of interest was all-cause mortality.

The study had ethical approval from the South Yorkshire Research Ethics Committee (REC ref: 12/YH/0344) and is conducted in accordance with ICH-GCP, Declaration of Helsinki, the Data Protection Act 1998 and NHS Act 2006.

### Statistical analysis

Continuous variables are expressed as median and interquartile range (IQR). Variables that were not normally distributed were log-transformed before analysis in further models. Categorical variables are expressed as numbers and percentages (%). Missing data were excluded from statistical analysis. Pearson’s Chi-squared test and Fisher’s exact row test were used to compare categorical variables between groups. Student’s *t *test and the Kruskal–Wallis test were used to compare continuous variables between the groups depending on the normality of the distribution.

Several Cox proportional hazard regression models were used to investigate the relation between variables and all-cause mortality. A first model was used to assign a weighting to the CXR variables based on their relation to prognosis. Only CXR variables were included in this model. Those significantly related to mortality on univariable analysis (a liberal threshold of *P* < 0.10 was used) were chosen for the multivariable model. The assigned points of the CXR score were generated by the beta coefficient (log hazard ratio) for each variable from the multivariable analysis, multiplied by ten and rounded to the nearest whole number [20].

A base model was constructed to assess the incremental value of the new CXR score in addition to other important clinical variables in predicting mortality. The base model included variables which had few missing data (< 1%) and which were also significantly related to mortality on univariable Cox regression analysis (again, a liberal threshold of *P* < 0.10 was used).

The individual CXR variables and the CXR score were then added to the base model, separately and in combination, to the find the best model to predict mortality. The new models’ cumulative discrimination was measured using Harrell’s C statistic; its statistical significance in comparison to the base model was assessed with the likelihood ratio test. A 2-sided *P* value ≤ 0.05 was considered statistically significant.

Venn diagrams are used to illustrate the relation between different features of pulmonary congestion. Kaplan–Meier curves, censored at 4 years, with the log-rank statistic were used to illustrate outcome.

Repeatability of CXR findings was tested using weighted Cohen’s Kappa statistic and Bland–Altman plots [21].

All analyses were performed using STATA software (version 14.2, StataCorp, Texas, United States) and Excel version 2016 (Microsoft, Redmond, United States).

## Results

### Participants

Between October 2012 and November 2016, 1145 patients were enrolled into OPERA-HF. After excluding 70 patients because they did not have a CXR during admission and another 100 patients because the CXRs were inadequate for interpretation, 975 remained (Fig. 1).

Table 1 shows the clinical and laboratory findings of patients recruited into the study on admission to hospital. Most patients had been hospitalised for HF in the previous year (60%). A quarter (26%) of patients did not have NT-proBNP measured on admission. Half of patients (54%) presented with breathlessness on exertion; 26% with breathlessness at rest and in the remainder, a symptom other than breathlessness was the reason for admission. 11% of patients presented with HF associated with an acute coronary syndrome.

**Table 1:** Clinical and laboratory findings of patients recruited into the study on admission to hospital

Variables	Patients	Missing
Demographics		
Age—years	77 (68–83)	0
Males	598 (61%)	
Hosp. for heart failure in the previous year	581 (60%)	
Prior myocardial infarction	220 (23%)	
Prior coronary artery bypass graft	136 (14%)	
Malignancy	108 (11%)	
Diabetes	323 (33%)	
Chronic obstructive pulmonary disease	160 (16%)	
ACS on admission for heart failure	101 (11%)	
Systolic blood pressure—mmHg	129 (114–147)	
Diastolic blood pressure—mmHg	74 (62–86)	
Main presenting symptom		
Breathlessness at rest	257 (26%)	0
Worsening breathlessness on exertion	529 (54%)	
Breathlessness not a main presenting symptom	189 (19%)	
Findings on electrocardiogram		
Atrial fibrillation	460 (47%)	28
QRS duration—milliseconds	104 (91–136)	
Heart rate—beats per minute	91 (73–112)	
Left ventricular systolic dysfunction on echocardiography		
None Mild Moderate Severe	233 (25%) 125 (14%) 218 (24%) 335 (37%)	64
Blood tests		
Sodium—mmol/L	137 (134–139)	0
Potassium—mmol/L	4.3 (4.0–4.7)	3
Urea—mmol/L	8.7 (6.2–13.0)	0
Creatinine—µmol/L	104 (82–144)	0
Troponin T—ng/ml	50 (29–148)	542
NT-proBNP—pg/ml	5047 (2337–10,945)	253
Haemoglobin—mmol/L	125 (110–139)	0
Chloride—mmol/L	101 (98–105)	
Albumin—mmol/L	34 (31–37)	2

28 patients had missing electrocardiograms because they could not be found in the clinical notes, 64 patients had missing echocardiograms because they had died before the investigation could be performed and 3 serum potassiums were missing because the blood samples had haemolysed

*QRS* duration–duration of QRS complex on electrocardiogram

*NT-proBNP* N-terminal-pro brain natriuretic peptide

Continuous variables are displayed as median (interquartile range) and categorical variables are displayed as number (percentage)

### Chest radiography appearances

Table 2 shows the CXR appearances of patients in the study. Most patients had AP CXRs (71%,). There was a low prevalence of features on the CXR unrelated to HF, including: consolidation (*N* = 91); pulmonary fibrosis (*N* = 16); hiatus hernia (*N* = 3); tumours/nodules (*N* = 7); pulmonary infarction (*N* = 5); pulmonary plaques (*N* = 13) and pneumonectomy (*N* = 1).

**Table 2:** Radiological findings of patients recruited into the study on admission, and in-hospital mortality, 30-day mortality and readmission to hospital within 30 days of discharge

Variables	Patients
Chest X-ray findings	
Cardiothoracic ratio (≤ 0.55)	261 (27%)
Cardiothoracic ratio (0.55–0.70)	606 (62%)
Cardiothoracic ratio (> 0.70)	53 (5%)
Cardiothoracic ratio: unmeasurable	55 (6%)
Cardiothoracic ratio	0.59 (0.55–0.64)
Film projection—anterior–posterior	691 (71%)
Cardiothoracic ratio (posterior-anterior films)	0.57 (0.53–0.61)
Cardiothoracic ratio (anterior–posterior films)	0.60 (0.55–0.64)
Moderate alveolar oedema	525 (54%)
Severe alveolar oedema	97 (10%)
Kerley B lines	688 (71%)
Pleural effusion	649 (67%)
Pulmonary venous congestion	756 (78%)
Outcomes *n* = (%)	
In-hospital mortality	41 (4%)
30-day mortality	44 (5%)
Readmission to hospital within 30-days of discharge	181 (19%)
All-cause mortality at end of follow-up	440 (45%)

Cardiomegaly was present in 67% and un-measureable in 6%. The median CTR (excluding those in whom a measurement was not possible, *N* = 55) was 0.57 (IQR 0.53–0.61) in those with PA films and 0.60 (IQR 0.55–0.64) in those with AP films. Pulmonary venous congestion was present in 78%, and a degree of alveolar oedema was present in 64% (although only 10% had severe alveolar oedema). Only one film had a pleural effusion that covered most of one lung field.

**Fig. 3:**
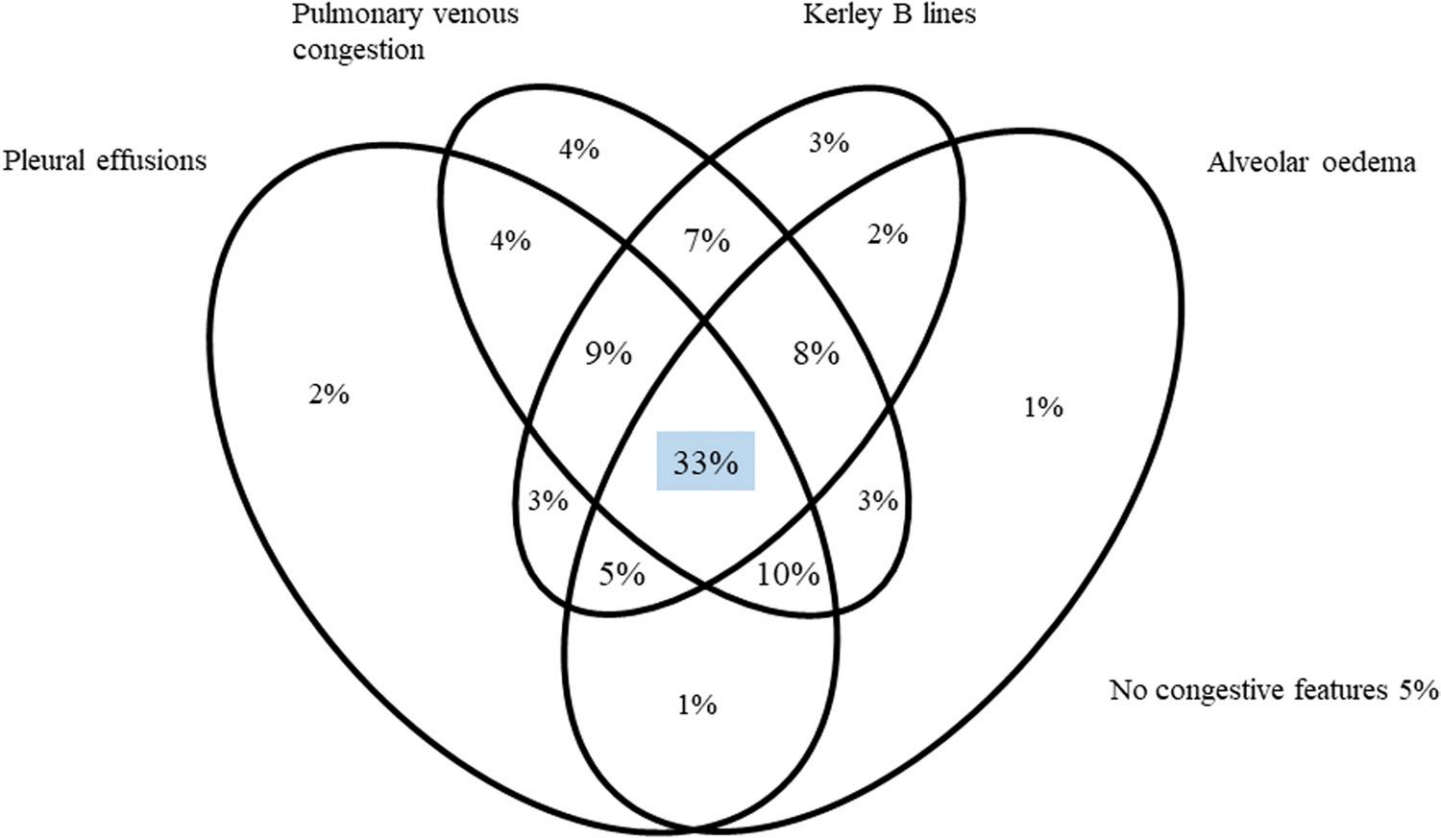
Venn diagram illustrating percentage of patients with different features of pulmonary congestion

**Fig. 4:**
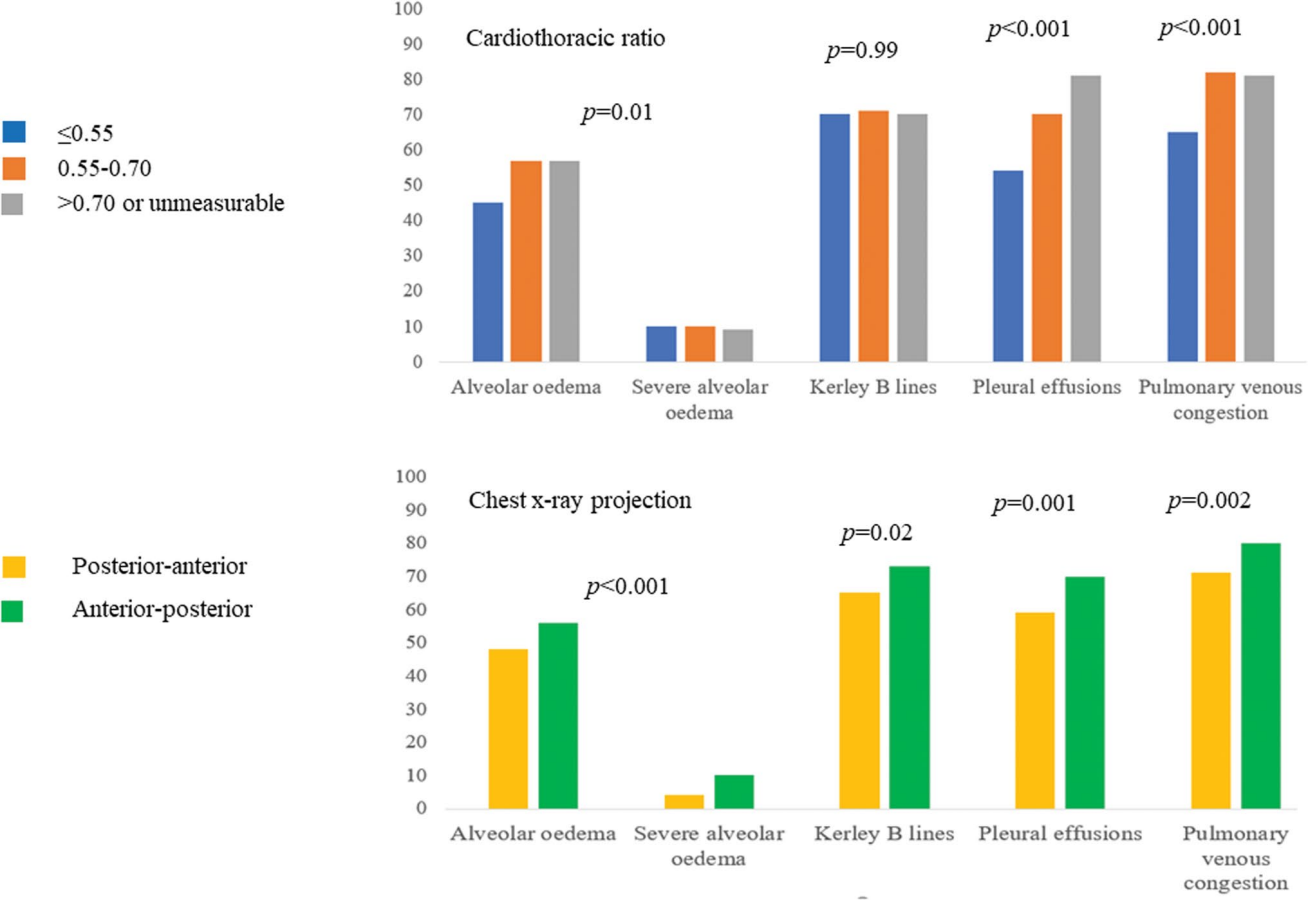
Bar charts illustrating proportion of patients with each feature of pulmonary congestion, by cardiothoracic ratio and chest X-ray projection. Pearson’s Chi-squared test was used

Figure 3 illustrates the percentage of patients with different features of pulmonary congestion. A third (33%) of patients had all four features of pulmonary congestion (Fig. 3); only 5% had none and 10% had only one. Features of pulmonary congestion were generally more common with increasing CTR and on AP compared to PA films as shown by the bar charts illustrating the proportion of patients with each feature of pulmonary congestion, by cardiothoracic ratio and chest X-ray projection in Fig. 4.

### Generation of the CXR score, relation to clinical variables and relation to outcome

After a median follow-up of 671 days, 440 (45%) patients had died of which 41 died during the index admission. A fifth (19%) of the patients was readmitted with worsening HF within 30 days of discharge from their index hospitalisation.

**Fig. 5:**
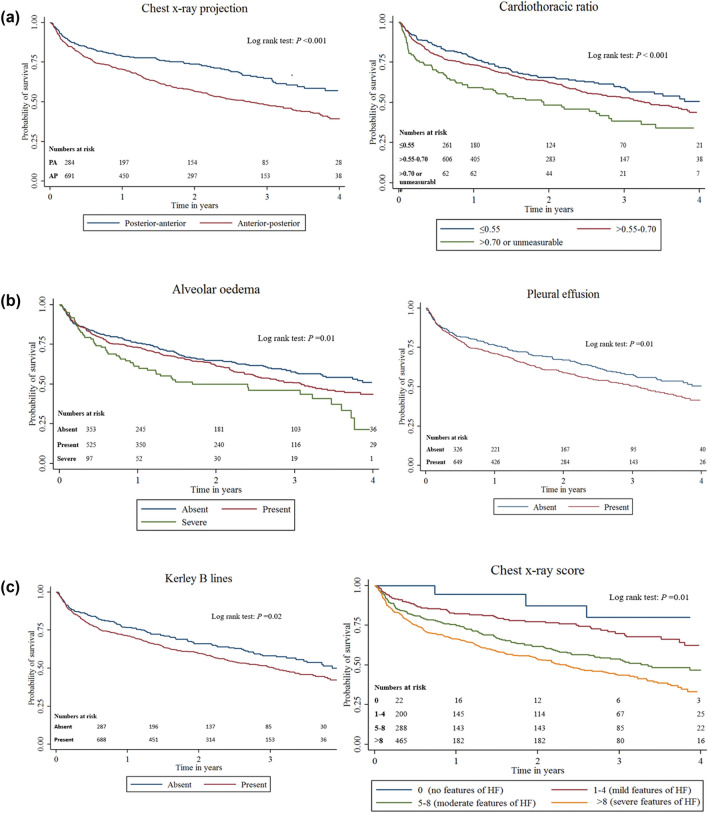
Kaplan–Meier curves of all-cause mortality for chest X-ray projection, cardiothoracic ratio, pleural effusion, alveolar oedema, Kerley B lines and the chest X-ray score. *AP* anterior–posterior, *PA* posterior–anterior, *HF* heart failure

Table 3 shows Cox regression analyses of chest X-ray variables and formation of the chest X-ray score; Fig. 5 shows Kaplan–Meier curves of all-cause mortality for chest X-ray projection, cardiothoracic ratio, pleural effusion, alveolar oedema. In univariable Cox regression analysis, each radiological abnormality, except pulmonary venous congestion, was associated with all-cause mortality. Consequently, a score was constructed using all CXR signs apart from pulmonary venous congestion.

**Table 3:** Univariable and multivariable Cox regression analyses of chest X-ray variables and formation of the chest X-ray score

Outcome: all-cause mortality	Hazard ratio (95% CI)	Wald *X*^2^	*P* value	Hazard ratio (95% CI)	β-coefficient	Score
Alveolar oedema						
Absent	Referent	Referent	Referent	Referent	Referent	0
Present	1.20 (0.98–1.47)	1.71	0.09	1.04 (0.84–1.31)	0.05	1
Severe	1.67 (1.22–2.28)	3.20	0.001	1.35 (0.97–1.88)	0.30	3
Kerley B lines						
Absent	Referent	Referent	Referent	Referent	Referent	0
Present	1.28 (1.04–1.59)	2.30	0.02	1.21 (0.98–1.51)	0.19	2
Cardiothoracic ratio						
≤ 0.55	Referent	Referent	Referent	Referent	Referent	0
0.55–0.70	1.21 (0.96–1.52)	1.62	0.10	1.12 (0.89–1.41)	0.12	1
> 0.70/unmeasurable	1.84 (1.35–2.50)	3.84	< 0.001	1.60 (1.16–2.19)	0.46	5
Chest X-ray projection						
Posterior-anterior	Referent	Referent	Referent	Referent	Referent	0
Anterior–posterior	1.13 (1.07–1.20)	4.28	< 0.001	1.47 (1.17–1.86)	0.38	4
Pleural effusions						
Absent	Referent	Referent	Referent	Referent	Referent	0
Present	1.29 (1.05–1.59)	2.47	0.01	1.12 (0.89–1.40)	0.11	1
Pulmonary venous congestion						
Absent	Referent	Referent	Referent	N/A	N/A	N/A
Present	1.06 (0.85–1.33)	0.53	0.60	N/A	N/A	N/A
Chest X-ray score						
Chest X-ray score	1.10 (1.07–1.13)	6.15	< 0.001	N/A	N/A	N/A

The score was constructed using the beta-coefficients (log hazard ratio) of a multivariable model, containing only chest X-ray variables that were significantly related to all-cause mortality on univariable analysis (*p* < 0.1). These variables were: alveolar oedema, Kerley B lines, cardiothoracic ratio, chest X-ray projection and pleural effusions. For example, the beta coefficient for Kerley B lines from the multivariate analysis is 0.19—which was rounded to 0.2 multiplied by 100 to give 2 points

*CI* confidence intervals, *N/A* not applicable

Table 4 shows clinical and laboratory findings of patients recruited into the study and in-hospital mortality, 30-day mortality and readmission to hospital within 30 days of discharge by increasing chest X-ray score. There was no relation between the CXR score and the presence (or absence) of left ventricular systolic dysfunction. Although associated with all-cause mortality at 4 years, the CXR score was neither associated with in-hospital mortality, nor mortality or readmission within 30 days of discharge. A worsening CXR score was associated with older age, higher serum concentrations of potassium and urea and higher plasma NT-proBNP (especially if the patient was in AF), lower values for diastolic blood pressure, haemoglobin, and albumin, being a woman, prior CABG and presenting with breathlessness as the main symptom.

**Table 4:** Clinical and laboratory findings of patients recruited into the study and in-hospital mortality, 30-day mortality and readmission to hospital within 30 days of discharge by increasing chest X-ray score

Variables	Chest X-ray score				*P* value
Demographics					
Chest X-ray score *N* = 975	0 *N* = 22 (2%)	1–4 *N* = 200 (20%)	5–8 *N* = 288 (30%)	> 8 *N* = 465 (48%)	
Age—years	74 (67–78)	73 (64–80)	76 (67–83)	78 (71–85)	< 0.001
Men	15 (68%)	148 (74%)	176 (61%)	259 (56%)	< 0.001
Hosp. for heart failure in the previous year	13 (59%)	111 (56%)	180 (63%)	277 (60%)	0.49
Prior myocardial infarction	6 (27%)	40 (20%)	64 (22%)	110 (24%)	0.71
Prior coronary artery bypass graft	7 (32%)	35 (18%)	39 (14%)	55 (12%)	0.02
Malignancy	3 (14%)	15 (8%)	36 (13%)	54 (12%)	0.27*
Diabetes	5 (23%)	57 (29%)	105 (36%)	156 (34%)	0.21*
Chronic obstructive pulmonary disease	4 (18%)	27 (14%)	41 (14%)	88 (19%)	0.22*
ACS on admission for heart failure	4 (18%)	12 (6%)	33 (12%)	52 (12%)	0.10*
Systolic blood pressure—mmHg	139 (113–154)	130 (115–146)	128 (116–150)	129 (112–145)	0.50
Diastolic blood pressure—mmHg	78 (65–86)	79 (65–89)	74 (63–87)	71 (62–85)	0.01
Breathlessness at presentation					
Not the main complaint	10 (46%)	34 (17%)	61 (21%)	84 (18%)	0.04*
Worsening on exertion	11 (50%)	113 (57%)	155 (54%)	250 (54%)	
Severe at rest	1 (5%)	53 (27%)	72 (25%)	131 (28%)	
ECG findings					
Atrial fibrillation	9 (41%)	98 (49%)	128 (44%)	225 (48%)	0.63
QRS duration—milliseconds	122 (34%)	113 (39%)	111 (33%)	118 (45%)	0.09
Heart rate—beats/minute	87 (25%)	93 (27%)	95 (31%)	96 (28%)	0.38
Left ventricular systolic dysfunction					
None—mild	8 (38%)	70 (36%)	97 (36%)	183 (43%)	0.21
Moderate—severe	13 (62%)	123 (64%)	174 (64%)	243 (57%)	
Blood tests					
Sodium—mmol/L	137 (135–139)	137 (134–139)	137 (134–139)	137 (134–139)	0.83
Potassium—mmol/L	4.3 (4.0–4.4)	4.3 (4.0–4.5)	4.3 (4.0–4.6)	4.4 (4.0–4.9)	0.02
Urea—mmol/L	9.3 (5.9–11.6)	8.1 (6.0–11.5)	8.3 (5.7–12.9)	9.4 (6.5–13.8)	0.002**
Creatinine—µmol/L	100 (90–156)	102 (81–129)	102 (81–145)	108 (83–154)	0.08**
Troponin T—ng/ml	36 (25–92)	40 (26–82)	53 (30–131)	59 (31–165)	0.08**
NT-proBNP—pg/ml	2079 (1467–2974)	4569 (1683–10,055)	5317 (2633–10,985)	5371 (2644–11,684)	0.003**
NT-proBNP in SR—pg/ml	2612 (1378–7211)	4999 (1899–10,510)	4987 (2628–12,031)	5498 (2185–11,793)	0.45**
NT-proBNP in AF—pg/ml	1826 (1467–2142)	3942 (1448–9599)	5663 (2633–10,682)	5307 (3117–11,684)	0.002**
Haemoglobin—mmol/L	136 (123–142)	130 (116–143)	122 (109–139)	123 (108–137)	0.004
Chloride—mmol/L	103 (100–105)	101 (98–105)	101 (98–105)	101 (97–105)	0.48
Albumin—mmol/L	37 (34–39)	34 (32–38)	34 (31–37)	34 (31–37)	0.002
Outcomes					
In-hospital mortality	0 (0%)	6 (3%)	11 (4%)	24 (5%)	0.57*
30-day mortality	0 (0%)	6 (3%)	13 (5%)	25 (5%)	0.52*
Readmission within 30 days of discharge	3 (14%)	34 (17%)	56 (20%)	88 (19%)	0.87*

*QRS* duration–duration of QRS complex on electrocardiogram, *NT-proBNP* N-terminal-pro brain natriuretic peptide

Continuous variables are displayed as median (interquartile range) and categorical variables are displayed as number (percentage). For categorical variables, Pearson’s Chi-squared test was used. If variables did not fit the assumptions of the Pearson’s Chi-squared test, Fisher’s exact test was used (labelled with *). For continuous variables, student’s *t *test and ANOVA was used to compare variables that were normally distributed and Kruskal–Wallis was used for variables that were not normally distributed (labelled with **)

CXR scores were lower in the 5% of patients (*n* = 49) whose first radiograph was taken more than 24 h after admission than in those who had their CXR earlier [median score 3 (IQR 3–7) v 7 (5–9), *P* < 0.001].

### Relation of the CXR score to prognosis in addition to other variables

Tables 5 and 6 show univariable and multivariable Cox regression analyses of clinical and laboratory variables and the chest X-ray score On univariable Cox regression analysis, age, gender, hospitalisation for heart failure in the previous year, prior MI or CABG, malignancy, lower systolic and diastolic blood pressures, lower heart rate, lower sodium, haemoglobin, chloride and albumin and higher potassium, renal function, troponin, and NT-proBNP, and a higher CXR score was related to mortality. In a multivariable model, older age and higher serum concentrations of urea and sodium, lower systolic blood pressure, lower heart rate and lower serum concentrations of chloride and albumin were independent predictors of mortality. The CXR score was also independently associated with mortality (HR 1.10, 95% CI 1.07–1.13 per point increase, *P* < 0.001). However, CXR score did not improve the model’s *c*-statistic for predicting mortality at different time-points as shown in Table 7.

**Table 5:** Univariable Cox regression analyses of clinical and laboratory variables and the chest X-ray score

Outcome: all-cause mortality	Univariable analysis		
	Hazard ratio (95% CI)	Wald *X*^2^	*P* value
Demographics			
Age (per year increase)	1.05 (1.04–1.06)	10.30	< 0.001
Gender (male vs female)			
Hosp. for heart failure in the previous year (yes vs no)	1.48 (1.21–1.80)	3.86	< 0.001
Prior myocardial infarction (yes vs no)	1.41 (1.14–1.74)	3..22	0.001
Prior coronary artery bypass graft (yes vs no)	1.55 (1.22–1.98)	3.52	< 0.001
Malignancy (yes vs no)	1.35 (1.01–1.80)	2.06	0.04
Diabetes (yes vs no)	1.19 (0.98–1.44)	1.74	0.08
Chronic obstructive pulmonary disease (yes vs no)			
ACS on admission for heart failure			
Systolic blood pressure—mmHg (per 10 unit increase)	0.93 (0.89–0.97)	– 3.71	< 0.001
Diastolic blood pressure—mmHg (per 10 unit increase)	0.98 (0.97–0.98)	– 7.29	< 0.001
Main presenting symptom			
Breathlessness not main presenting symptom	Referent	Referent	Referent
Worsening breathlessness on exertion	1.05 (0.83–1.35)	0.43	0.67
Breathlessness at rest	0.82 (0.62–1.09)	– 1.38	0.17
Findings on ECG			
Atrial fibrillation (yes vs no)	1.07 (0.89–1.29)	1.70	0.49
QRS duration—milliseconds (per unit increase in log QRS duration)	1.13 (0.82–1.56)	0.75	0.45
Heart rate—beats/second (per 10 unit increase)	0.92 (0.88–0.95)	– 4.92	< 0.001
Left ventricular systolic dysfunction			
None or mild	Referent	Referent	Referent
Moderate or severe	0.78 (0.64–0.95)	– 2.42	0.02
Blood tests			
Sodium—mmol/L (per unit increase)	0.97 (0.95–0.99)	– 2.64	0.008
Potassium—mmol/L (per unit increase)	1.20 (1.04–1.39)	2.51	0.01
Urea—mmol/L (per unit increase in log urea)	2.35 (2.00–2.77)	10.26	< 0.001
Creatinine—μmol/L (per unit increase in log creatinine)	2.33 (1.90–2.86)	8.06	< 0.001
Troponin—ng/ml (per unit increase in log troponin)	1.17 (1.08–1.27)	3.68	< 0.001
NT-proBNP—pg/ml (per unit increase in log NT-proBNP)	1.33 (1.20–1.48)	5.43	< 0.001
Haemoglobin—mmol/L (per 10 unit increase)	0.88 (0.84–0.92)	– 6.15	< 0.001
Chloride—mmol/L (per 10 unit increase)	0.61 (0.51–0.73)	– 5.49	< 0.001
Albumin—mmol/L (per 10 unit increase)	0.60 (0.50–0.72)	– 5.37	< 0.001
Chest X-ray score			
Chest X-ray score (per unit increase)	1.10 (1.07–1.13)	6.15	< 0.001

Variables which were not normally distributed were log-transformed

*QRS* duration–duration of QRS complex on electrocardiogram, *NT-proBNP* N-terminal-pro brain natriuretic peptide, *N/A* not applicable

**Table 6:** Multivariable Cox regression analysis

Outcome: all-cause mortality	Multivariable analysis (base model)			Multivariable analysis (base model and chest X-ray score)		
	Hazard ratio (95% CI)	Wald *X*^2^	*P* value	Hazard ratio (95% CI)	Wald *X*^2^	*P* value
Demographics						
Age (per year increase)	1.04 (1.03–1.05)	6.95	< 0.001	1.04 (1.03–1.05)	6.33	< 0.001
Systolic blood pressure—mmHg (per 10 unit increase)	0.94 (0.89–0.99)	− 2.52	0.01	0.94 (0.90–0.99)	− 2.40	0.02
Findings on ECG						
Heart rate—beats/minute (per 10 unit increase)	0.95 (0.91–0.98)	− 2.78	0.005	0.94 (0.90–0.97)	-3.25	0.001
Blood tests						
Sodium—mmol/L (per unit increase)	1.05 (1.01–1.08)	− 2.55	0.01	1.04 (1.01–1.08)	2.33	0.02
Urea—mmol/L (per unit increase in log urea)	1.41 (1.01–1.98)	2.00	0.05	1.42 (1.01–1.99)	2.01	0.04
Chloride—mmol/L (per 10 unit increase)	0.51 (0.38–0.67)	− 4.69	< 0.001	0.51 (0.38–0.67)	− 4.71	< 0.001
Albumin—mmol/L (per 10 unit increase)	0.69 (0.54–0.88)	− 3.03	0.002	0.71 (0.56–0.91)	− 2.72	0.006
Chest X-ray score						
Chest X-ray score (per unit increase)	N/A			1.07 (1.03–1.11)	3.75	< 0.001

Only variables significantly related to prognosis (*p* < 0.05) are displayed. Variables which were not normally distributed were log-transformed. The base model contains only variables that were significantly related to all-cause mortality on univariable analysis, displayed in Table 3

*QRS* duration–duration of QRS complex on electrocardiogram, *NT-proBNP* N-terminal-pro brain natriuretic peptide, *N/A* not applicable

**Table 7:** Reproducibility statistics (Cohen’s kappa for categorical variables and Bland Altman limits of agreement for continuous variables) got the chest X-ray variables and the chest X-ray score as well as the Harrell’s *c*-statistic and likelihood ratio tests of the multivariable Cox regression models

Model	Reproducibility (*p* value/95% CI)	Harrell’s concordance statistic (*P* value)			
Mortality (*N*, %)		1 month (38, 100%)	1 year (263, 80%)	3 years (409, 56%)	4 years (435, 48%)
Base model		0.84	0.72	0.70	0.71
+ B lines	0.55 (0.001)	0.84 (0.42)	0.72 (0.55)	0.70 (0.90)	0.71 (0.76)
+ Effusions	0.78 (< 0.001)	0.84 (0.90)	0.72 (0.14)	0.70 (0.39)	0.71 (0.83)
+ Oedema	0.82 (< 0.001)	0.85 (0.10)	0.72 (0.05)	0.70 (0.18)	0.71 (0.30)
+ CTR	0.53 (0.003)	0.84 (0.65)	0.72 (0.01)	0.70 (0.02)	0.71 (0.01)
+ Projection	N/A	0.83 (0.51)	0.72 (0.01)	0.70 (0.19)	0.71 (0.05)
+ CXR score	0.40 (-1.90–2.67)	0.84 (0.77)	0.72 (< 0.001)	0.70 (0.01)	0.71 (0.006)

The base model contains variables displayed in Tables 5 and 6 and is the same base model used in this table

*CI* confidence intervals, *N/A* not applicable

### Reproducibility

CXR variables varied in reliability, with alveolar oedema being the most reliable and CTR being the least, as shown in Table 7.

## Discussion

There are three main findings from this study. First, radiological evidence of congestion is very common in patients presenting to hospital with HF. Second, patients presenting with breathlessness as their dominant symptom have a higher CXR score. Third, increasing pulmonary congestion on the CXR score is related to worsening HF as assessed by other clinical measures such as age, potassium, urea, NT-proBNP, haemoglobin and albumin, and is associated with increasing all-cause mortality, but not related to overall prognosis when these other variables are taken into account.

We found that the commonest abnormal feature on the chest X-ray was venous congestion, presumably representing a rise in left atrial pressure. As heart failure worsens, fluid can escape from pulmonary capillaries into the interstitial and pleural spaces resulting in Kerley B lines and pleural effusions, the next two most common features we found. Frank alveolar oedema was the least common finding, and represents the patient passing a ‘tipping point’ when the capacity of the lymphatics to remove fluid is exceeded and fluid starts to accumulate in the airspaces of the lungs [16].

Previous older studies have graded congestion in an incremental fashion based on the aforementioned sequence of events, with stage one being pulmonary venous congestion, stage two interstitial oedema and stage three alveolar oedema [11–13, 22]. We chose to grade all features separately and found that although many had pulmonary venous congestion and few had severe alveolar oedema, patients commonly have features of pulmonary congestion not in sequence. It may be that if pulmonary congestion happens very rapidly, the radiographic features might not occur in order.

Our study findings confirm that the CXR can identify pulmonary congestion as a potential cause of breathlessness in patients presenting to hospital. Since congestion on the CXR is still related to mortality in HF patients on univariable analysis, this should prompt clinicians to adequately diurese the patient when necessary.

Only a small proportion of patients (91/975, 9%) in our cohort had evidence of pneumonia on the CXR. Future studies should investigate whether the CXR can help to differentiate pulmonary congestion from other causes of breathlessness, and influence therapeutic decision making beyond HF, such as the initiation of antibiotics. The CXR remains a source of important clinical information and may be used within artificial intelligence algorithms, to help assist clinicians improve the diagnosis of AHF and guide management [23].

To our knowledge, this is the first study which has found a relation between CXR projection and all-cause mortality. If a patient is too unwell to stand, inhale and hold their breath, then an AP film in the sitting upright position is performed [24], and thus projection is a marker of a patient’s fitness during their acute illness.

Our findings agree with previous work, which found that cardiomegaly on the CXR, regardless of projection, was related to a worse prognosis in patients with acute and chronic HF [9, 10]. However, despite a high level of readmission to hospital within 30 days of discharge, in-hospital and 30-day mortality was low. None of these outcomes strongly related to the degree of congestion, consistent with the observation that early readmissions are often not for HF [25–27]. Our CXR score was strongly related to clinical variables that are related to the prognosis of patients with acute HF. Thus, it is not surprising that CXR features do not add much to the predictive power of models that included these variables.

One surprising finding was that a lower initial heart rate was related to higher mortality. Lourenco and colleagues had similar findings amongst 564 patients presenting with acute HF to a single centre [28]. In both studies, the first heart rate measurement was recorded on presentation to hospital. Although a higher resting heart rate predicts higher mortality in chronic HF, a faster initial heart rate in acute HF may be a marker of a preserved autonomic nervous response.

### Limitations

This is a single centre study. Most patients were of Caucasian ethnicity. Notably, Kobayashi and colleagues recently examined pulmonary congestion on the CXR in 117 patients and found that a worsening congestion score index was associated with a composite of all-cause mortality or rehospitalisation for HF at 90 days [29]. The index was generated from dividing the chest X-ray into six zones, and grading the severity of congestion in each lung zone. Our method of grading the CXR is far more likely to be the way CXRs are routinely examined in the UK. We have also taken into account radiograph projection or CTR, both of which we have shown to relate to worse prognosis.

Our version of the CXR score need to be validated in another population. Although reports from radiologists were used, no radiologists were involved in the retrospective review of the films. This may account for some of the variation in reproducibility of CXR variables and possibly misinterpretation of films, such as pneumonia being mistaken for localised alveolar oedema. However, diagnosis and treatment of HF is started as soon as the patient is admitted to hospital and not usually after radiology consult. The Heart Failure Association of the European Society of Cardiology has published a position paper which recommends proper assessment of cardiomegaly, pulmonary venous congestion, pleural effusions, interstitial and alveolar oedema by all admitting physicians [30]. This study, therefore, reflects how CXRs would be interpreted by the majority of clinicians treating patients presenting with breathlessness.

Most CXRs were done within the first 24 h of admission but the CXR scores of those who had their first film after 24 h were lower. It is unknown which of these CXR appearances were influenced by diuretic administration. In another study, Kobayashi and colleagues found that residual pulmonary congestion on the CXR prior to discharge predicted a composite outcome of all-cause mortality or rehospitalisation for heart failure at 1 year [31].

## Conclusions

In patients admitted to hospital for HF, there is a high prevalence of radiographic features of congestion. Worsening features of congestion are associated with variables related to worse prognosis. An increase in the CXR score is related to increasing all-cause mortality but is not an independent predictor of outcome when other variables are taken into account.

## Data Availability

Available from corresponding author (DP) upon reasonable request.
